# Re-Do Plastic Reconstruction for Locally Advanced and Recurrent Colorectal Cancer Following a beyond Total Mesorectal Excision (TME) Operation—Key Considerations

**DOI:** 10.3390/jcm13051228

**Published:** 2024-02-21

**Authors:** Emmanuel Giannas, Konstantinos Kavallieros, Theodoros Nanidis, John Giannas, Paris Tekkis, Christos Kontovounisios

**Affiliations:** 1 Department of Surgery and Cancer, Imperial College London, London SW7 2BX, UK; emmanouil.giannas18@imperial.ac.uk (E.G.); konstantinos.kavallieros19@imperial.ac.uk (K.K.); p.tekkis@imperial.ac.uk (P.T.); 2 Department of General Surgery, Chelsea and Westminster Hospital, London SW10 9NH, UK; 3 Department of Plastic Surgery, The Royal Marsden Hospital, London SW3 6JJ, UK; theodore.nanidis@rmh.nhs.uk; 4 Department of Plastic and Reconstructive Surgery, Euroclinic, 115 21 Athens, Greece; john.giannas@gmail.com; 5 Department of Plastic and Reconstructive Surgery, The London Welbeck Hospital, London W1G 83N, UK; 6 Department of Surgery, The Royal Marsden Hospital, London SE3 6JJ, UK; 7 2nd Surgical Department Evaggelismos, Athens General Hospital, 115 21 Athens, Greece

**Keywords:** reconstruction, pelvic exenteration, colorectal cancer, recurrent, beyond TME

## Abstract

Innovation in surgery and pelvic oncology have redefined the boundaries of pelvic exenteration for CRC. However, surgical approaches and outcomes following repeat exenteration and reconstruction are not well described. The resulting defect from a second beyond Total Mesorectal Excision (TME) presents a challenge to the reconstructive surgeon. The aim of this study was to explore reconstructive options for patients undergoing repeat beyond TME for recurrent CRC following previous beyond TME and regional reconstruction. MEDLINE and Embase were searched for relevant articles, yielding 2353 studies. However, following full text review and the application of the inclusion criteria, all the studies were excluded. This study demonstrated the lack of reporting on re-do reconstruction techniques following repeat exenteration for recurrent CRC. Based on this finding, we conducted a point-by-point discussion of certain key aspects that should be taken into consideration when approaching this patient cohort.

## 1. Introduction

Colorectal cancer (CRC) presents a significant healthcare burden; in the United Kingdom, over 14,000 patients are diagnosed annually [1]. An estimated 10% of these are locally advanced at presentation and require management beyond Total Mesorectal Excision (TME) planes [1]. Furthermore, the COVID-19 pandemic has disrupted the delivery of surgical care worldwide, with predicted increases in the proportion of patients presenting with more advanced cancer [2]. In complex CRC, (i.e., locally advanced or recurrent), pelvic exenteration followed by regional reconstruction is commonly performed. The aim of this procedure is to achieve clear resection margins (R0). This has been established as the most significant predictive factor for survival and quality of life (QoL) in complex CRC [1,3,4,5]. 

In recent decades, innovations in surgery and pelvic oncology have redefined the boundaries of pelvic surgery [5]. Pelvic exenteration has evolved to be safe and feasible, and therefore the definitive treatment for complex CRC [6]. The estimated 5-year survival following exenteration procedures is up to 65% [7]. However, a portion of patients having undergone pelvic exenteration will have local recurrence, either due to residual microscopic disease progression or insufficient primary resection margins. In some patients, recurrent disease may be amenable to repeat exenteration, followed by re-do reconstruction. 

However, surgical reconstructive approaches and outcomes following repeat exenteration are not well described. It is nevertheless expected that morbidity following further visceral resection is high. One of the main challenges is managing to adequately reconstruct the pelvic region. The resulting defect from a second beyond TME procedure invariably presents a challenge to the reconstructive surgeon. The surgical options are more limited than in primary reconstruction, as most patients will have had some degree of reconstruction during their first exenteration, with the reconstructed sites being irradiated, or potentially requiring resection due to local recurrence [8]. The aim of this study was to explore reconstructive options for patients undergoing repeat exenteration for recurrent CRC following previous pelvic exenteration and regional reconstruction. We conducted a review of the literature and performed a point-by-point discussion of key consideration that should be taken into account during surgical planning for these complex patients. 

## 2. Materials and Methods

### 2.1. Literature Search 

**Figure 1:**
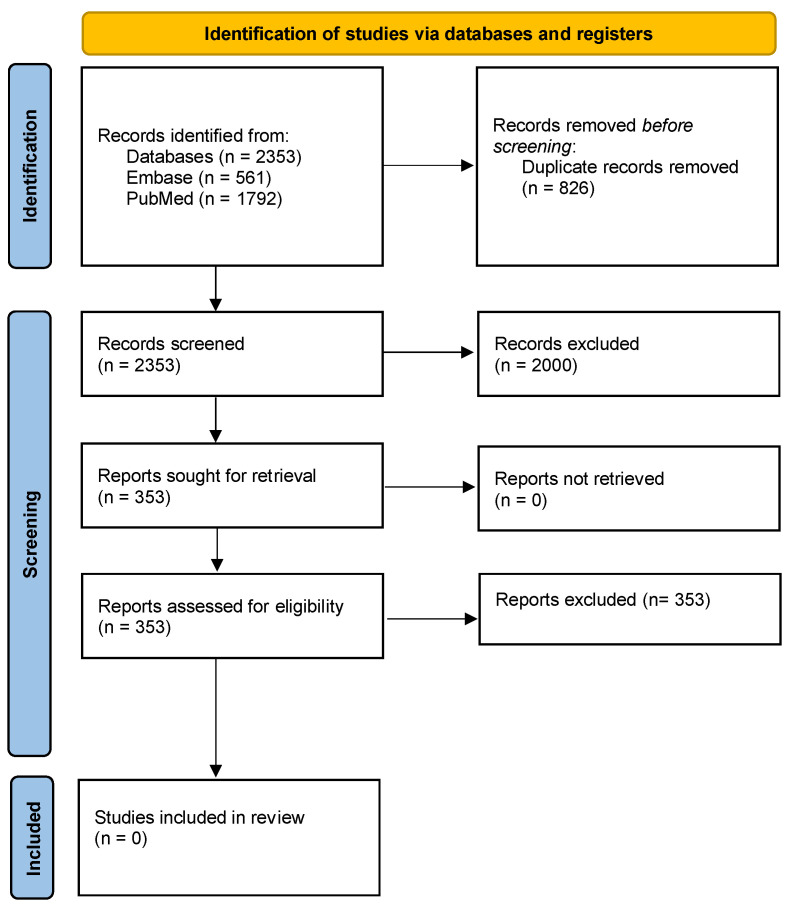
PRISMA flowchart.

This study was conducted in accordance with the Preferred Items for Systematic Reviews and Meta-Analysis (PRISMA) guidelines (Figure 1 and Supplementary Materials) [9]. A focused review of the literature was performed under the guidance of a qualified medical librarian to ensure a robust search strategy. MEDLINE (PubMed) and EMBASE databases were used to search for relevant articles. The last day for this search was 11 August 2023. 

### 2.2. Search Strategy

Research articles from 1990 onwards were considered. A combination of MeSH terms and keywords was used to produce the search strategy. To identify studies reporting on patients undergoing pelvic exenteration, we combined the MeSH term “Pelvic Exenteration” with the keywords “pelvic exenteration” and “pelvic evisceration”. To identify studies reporting on patients with recurrent colorectal disease who underwent regional reconstruction, the MeSH term “recurrence” was combined with the keywords “recurrence”, “reconstruction”, and “redo reconstruction”. Finally, to identify studies reporting on cancer, the MeSH term “cancer, pelvic” was used. 

### 2.3. Inclusion Criteria

Inclusion required the studies to report on secondary reconstruction following recurrent CRC disease curative resection and provide information on the primary reconstructive technique. No minimum follow up was required for study inclusion. Case reports, cohort, comparative, and randomized control studies were considered. Only studies conducted in the English language using human adult subjects were included. 

### 2.4. Exclusion Criteria

Studies reporting only on the outcomes following primary reconstruction post pelvic exenteration were not considered. In addition, studies that did not provide details of both the primary and re-do reconstruction following the second beyond TME were excluded. Lab and animal studies were excluded. Abstracts, editorial correspondence, book chapters, and non-English articles were not considered. 

### 2.5. Citation Management

Two independent reviewers performed title and abstract screening. Any disagreements were discussed and resolved with the senior author in group meetings. A full text review was performed by two authors independently. Studies that could not be retrieved were requested and provided by the medical library team. COVIDENCE Version 2.0 (Melbourne, Australia) and Microsoft Excel Version 16.81 (Redmond, WA, USA) were used to screen articles and manage citations. 

## 3. Results

Following deduplication, our search strategy yielded 2353 studies. Title and abstract screening resulted in 353 studies for full text review. However, following full text review, all the references were excluded, making it impossible to conduct a review on re-do reconstruction options following repeat exenteration for recurrent CRC. No studies met the inclusion criteria as they did not provide details on the primary and re-do reconstruction. None of the studies reported on primary and re-do reconstructive options or outcomes following a second beyond TME operation. Similarly, studies that reported on reconstruction following recurrent CRC disease and exenteration did not provide details on either the primary reconstruction or the re-do reconstruction.

## 4. Discussion

The main finding of this review was that there is a lack of reporting on re-do reconstruction techniques following repeat pelvic exenteration for recurrent or locally advancing CRC. With the increasing incidence of repeat exenterations, patients require more complex reconstruction and in several cases re-do reconstruction. The current lack of reporting on this cohort of patients is concerning. Based on this finding, we have conducted a point-by-point discussion of certain key aspects that we think that should be taken into consideration when approaching this cohort of patients. 

### 4.1. Consideration 1—Pre-Operative Planning

Re-do reconstruction following repeat pelvic exenteration is very challenging. For negative margin resection in repeat surgery, the surgeon may have to resect “higher and wider” [1]. This necessitates detailed preoperative preparation and patient optimisation. To achieve this, a multidisciplinary team (MDT) approach is recommended, which requires a team with surgical, oncological, radiological, pathological, and reconstructive expertise [10]. This will have to include surgeons, anaesthetists, radiologists, specialist nurses, and physicians including medical oncologists and radiation oncologists. Discussion should include the potential for repeat exenteration and reconstruction, in light of the increasing incidence of recurrent disease, especially in younger patients. 

Each individual case in this group of patients is unique and therefore necessitates a collaborative approach. If during surgical planning the forecasted defect is potentially deemed unreconstructable, then the feasibility of the whole procedure should be reassessed. In these complex cases, it is unlikely that only one mode of reconstruction would be enough and generally a combination of flaps may be required. An assessment of the viability of the initial reconstruction and its potential for re-harvesting and subsequent utilization alongside a secondary flap is critical. If the initial flap lacks suitability for re-harvesting, the next feasible option involves regional flaps, followed by free flaps as a last resort. Overall, the need for an experienced reconstructive surgeon to facilitate these early planning discussions is vital to ensure optimal patients outcomes. 

### 4.2. Consideration 2—Pre-Operative Imaging Evaluation Using Computed Tomography Angiography (CTA) and Magnetic Resonance Imaging (MRI)

Different strategies for preoperative imaging investigations of patients with re-recurrent CRC have been described. Given the technically challenging nature of the operation, thorough preoperative imaging is important in determining the surgical and reconstructive plan. An important consideration for reconstruction based on tissue flaps is the variable anatomy of perforator locations, including their position and diameter [11]. CTA has been shown to be the gold standard in perforator imaging, particularly in autologous breast reconstruction [12]. Recent studies have demonstrated the utility of CTA in pelvic reconstruction. CTA can be useful for visualising deep inferior epigastric arteries (DIEA) and perforator vessels, providing useful pre-operative information helping guide flap selection for perineal reconstruction following extensive pelvic surgery [11,12]. Additionally, the patients undergoing repeat exenteration will often have had pelvic reconstruction following the initial exenteration. Post-operative evaluation of primary pelvic reconstruction has been well documented. Recurrent CRC may be difficult to differentiate from postoperative changes in flap placement and postoperative anatomic distortion. MRI is commonly used in preference to CT; particular consideration should be given to changes depicted in diffusor-weighted imaging (DWI), combined with specific features depicted by conventional MR sequences (T1, T2, fsT1+C), which can aid in tumour vs. normal flap differentiation [13]. 

Sarcopenia is a recognized risk factor for post-operative complications—including skin flap necrosis—and increased morbidity [14,15]. The routine use of sarcopenia measuring software can be used to identify patients at increased risk, with relatively low additional time and cost [16]. These software models could potentially be used to pre-operatively assess optimal sites to raise a flap. In addition, special considerations could be made for post-operative monitoring of this sub cohort of patients, including dedicated physiotherapy and plastic specialist nurses, as their flaps are at increased risk of failure [16]. 

### 4.3. Consideration 3—Routine Reconstruction Options 

**Figure 2:**
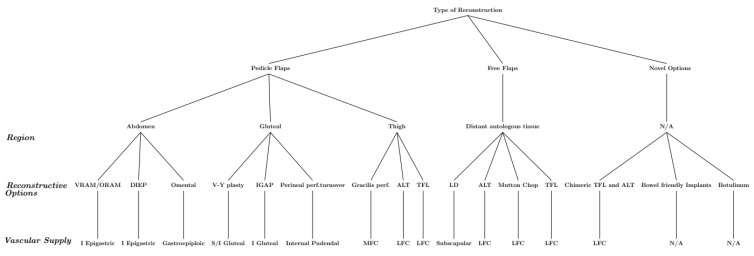
Flowchart of the reconstructive options for re-do reconstruction in patients undergoing repeat pelvic exenteration for CRC. VRAM: Vertical Rectus Abdominis Myocutaneous, ORAM: Oblique Rectus Abdominis Myocutaneous, DIEP: Deep Inferior Epigastric Perforator, IGAP: Inferior Gluteal Artery Perforator (IGAP), ALT: Anterior Lateral Thigh, TFL: Tensor Fascia Latae, LD: Latissimus Dorsi, I Epigastric: Inferior Epigastric, S Gluteal: Superior Gluteal, I Gluteal: Inferior Gluteal, MFC: Medial Femoral Circumflex, LFC: Lateral Femoral Circumflex, N/A: Not applicable.

**Figure 3:**
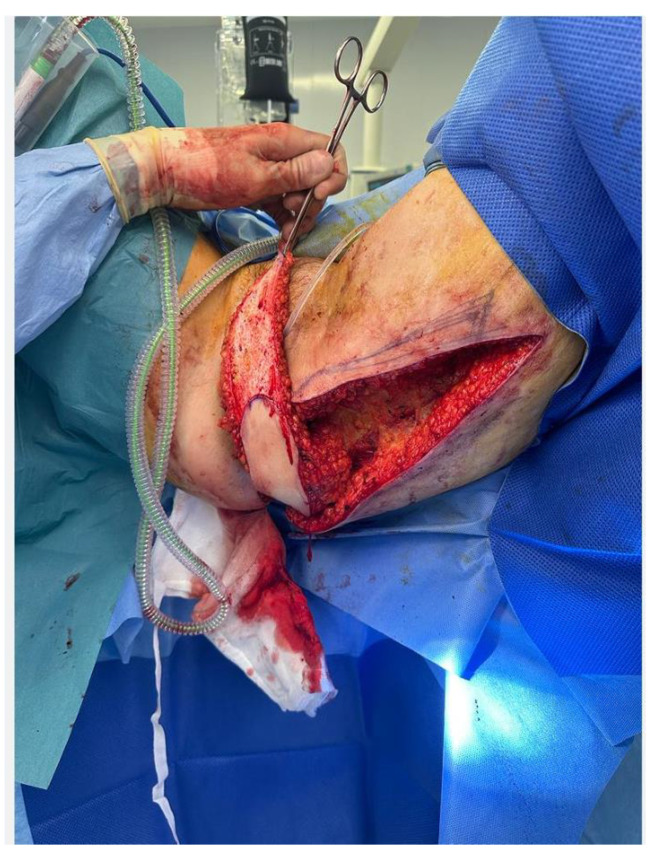
Gluteal fold perforator flap for reconstruction of pelvic defect.

**Figure 4:**
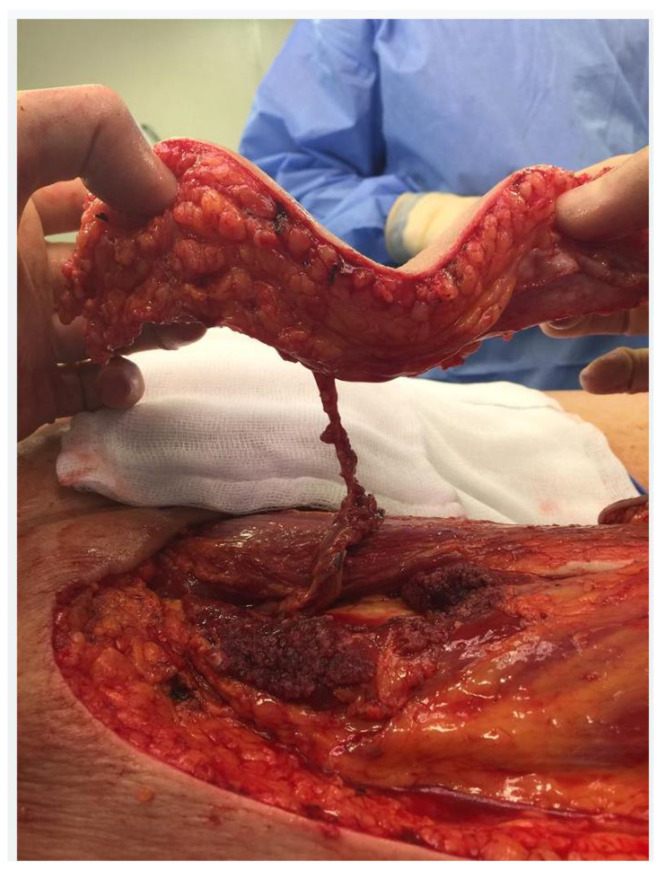
Harvested Anterior Lateral Thigh pedicle. The flap has been tunnelled under the rectus femoris muscle to improve its reach. Another option to extend the reach of the ALT is to extend its pedicle with cable vascular grafts: either a vein graft or the deep inferior epigastric vessels or the contralateral ALT pedicle.

Patients undergoing repeat exenteration often require repeat reconstruction due to extensive soft tissue loss, which may include the site of primary reconstruction. Regional and surgical factors such as degree of primary and secondary exenteration, choice of primary reconstruction, pelvic dead space, and regional vascular supply have a significant role in determining which reconstructive options are available [11,17,18]. The main soft tissue reconstruction options that are most considered can be grouped into three categories: abdominal, gluteal, and thigh [1,17,18,19] (Figure 2). The abdominal pedicles that may be available are the vertical or oblique rectus abdominis myocutaneous (VRAM/ORAM) and the deep inferior epigastric perforator (DIEP) [1,17,18,20]. The gluteal pedicles include the inferior gluteal perforator (IGAP), myocutaneous or fascio-cutaneous V-Y plasty, and the internal pudendal artery perforator or perineal turnover flap [1,17,18] (Figure 3). The thigh options include the anterior lateral thigh (ALT) and bilateral pedicled gracilis flaps (Figure 4) [1,17,18,19]. However, in patients with repeat exenteration requiring re-do reconstruction, these options may be severely restricted and are not typically available. Given the increasing incidence of repeat exenterations, care must be taken when planning primary exenteration and reconstruction to attempt to maintain as many reconstructive options as possible in case future exenterations with reconstruction are needed. Potential examples of how this could be achieved include determining the feasibility of a hybrid model combining the use of a mesh and autologous tissue. Therefore, instead of doing two autologous reconstructions, a flap and mesh can be used for the primary reconstruction and the second flap preserved with another mesh in case a re-do reconstruction is needed in the future. 

In certain cases, regional spread to the iliac vessels requires en bloc resection to achieve R0 margins. In these cases, not only do the iliac vessels need to be reconstructed, but regional reconstructive options which are dependent on the iliac vessels can no longer be used.

Another regional reconstructive option is the tensor fasciae latae (TFL) myocutaneous flap that has been described by Russel et al. [21]. There are several advantages with this flap, as it is simple to harvest, versatile, and has the potential to include bone. Furthermore, due to its anatomical location, the TFL can be used a as pedicle flap for intra-abdominal and pelvic defects without the need for free tissue transfer [21,22]. 

A common complication following extensive exenteration is empty pelvic syndrome (EPS), which contributes to significant post-operative morbidity [23]. The pathophysiology underlying empty pelvic syndrome is related to the degree of soft tissue reconstruction. It is thought that it usually develops in cases with inadequate intra-abdominal coverage. This results in pockets where fluid and bacteria can collect, resulting in wound dehiscence, abscess formation, and in some cases sepsis [23]. Despite its clinical significance, there remains a lack of comprehensive research addressing effective strategies for managing EPS. This highlights an ongoing unmet need in the management of these patients, where further research is required to develop approaches aiming to improve the prevention and management of EPS [24]. Patients undergoing re-do reconstruction may be at higher risk of developing EPS-related complications due to extensive resection and challenging reconstruction. A recent protocol published by the PelvEx collaboration has outlined their attempts to determine core outcome and descriptor sets that are measurable and will allow a consensus to be established to reduce research heterogeneity [24]. Reconstructive techniques that have been described to attempt to reduce this phenomenon include omental flaps and the use of mesh [23,25,26,27]. In certain cases where viable myocutaneous flaps were not available, a combination of omental flap with acellular dermal matrix has been used. This combination may be a useful option to provide structural support [17,26]. 

### 4.4. Consideration 4—Free Flaps 

Once regional reconstructive options have been exhausted, free tissue transfer will be the only remaining viable autologous tissue option. The free latissimus dorsi (LD) has been described in the literature for pelvic and lower limb reconstruction [28,29]. The anatomical features of the LD allow for multiple variations in flap harvesting, which in some cases can even allow for the preservation of upper limb function [28]. A free LD flap has, therefore, several advantages which include a large surface area, ease of harvesting, and a good stable circulation. However, care must be taken when identifying pelvic vessels for anastomosis, as the extensive exenteration and radiated tissue may have distorted regional anatomy, which can make vessel identification and anastomosis challenging [29]. In certain cases, a subtotal ALT free flap may be an option, using a saphenous arteriovenous loop by anastomosing the distal saphenous vein to the superficial femoral artery [30]. The loop is then transected at its centre in order to provide arterious and venous recipient vein grafts [30]. The ALT flap can include one or more muscles, such as the vastus lateralis or rectus femoris, depending on the amount of dead space [30]. The “Mutton Chop” flap is a musculocutaneous free flap based on the lateral circumflex femoral artery described by Vogt et al. [20]. This includes a large anterior skin island, fascia, rectus femoris, sartorius, and the tensor fasciae latae, providing a valuable option in patients with extensive pelvic defects where regional options are not available [20]. 

### 4.5. Consideration 5—Novel Reconstructive Options

Novel approaches to reconstruct pelvic and abdominal wounds have been proposed in the past. These approaches may be particularly useful in the cohort of patients examined in this study. One example is the use of a chimeric TFL and ALT flap. In particular, the TFL and ALT flap can be harvested together with a lower-down skin paddle, which might provide adequate coverage and therefore be a useful option.

Carboni et al. have described their experience with the use of breast prosthesis to reconstruct the pelvic floor following pelvic exenteration. They used an anatomical shaped silicone breast implant which adapts to the regional pelvic anatomy, aiming to reduce empty pelvic syndrome post-exenteration and reduce morbidity [31]. With this technique they reported a complication rate of 37.5%, with only two patients from a cohort of fifty-three requiring implant removal. Interestingly, no side effects were reported from the 16 patients requiring adjunctive radiotherapy [31]. Therefore, they suggested that the main advantages of the use of bowel-friendly implants are that it minimizes complications from empty pelvic syndrome in patients with limited reconstructive options but also may be used to exclude the bowel from the radiation field.

Botulinum toxin injections are increasing being used in abdominal wall reconstruction (AWR) [32]. Data suggest that bilateral pre-treatment in hernia patients significantly reduces lateral abdominal wall tension, increasing muscle volume and therefore facilitating the midline closure of large abdominal defects [32]. This concept could provide an interesting option for re-do reconstruction in pelvic exenteration patients. 

### 4.6. Consideration 6—Lexicon Collaboration

The heterogeneity of operative description of pelvic exenteration techniques has been recognised in the literature [33]. In this context, the Lexicon Collaboration has developed a validated “Pelvic Exenteration Lexicon” to promote the accurate description and classification of surgical techniques [33]. This aims to improve communication between surgical teams, enable more accurate data collection, and hence allow for more accurate comparisons between different techniques and procedures. Further development of the “Pelvic Exenteration Lexicon” to include a more detailed description of reconstructive options in pelvic exenteration patients may improve outcome monitoring. Furthermore, it is important to examine the pathophysiology and how reconstructive approaches may contribute to EPS-related complications, since they are associated with significant post-operative mortality and morbidity, as highlighted recently by the PelvEx collaboration [24]. Therefore, future work should aim to explore the prevalence of EPS in re-do reconstruction and determine how to minimize its complication rate in this complex cohort of patients. 

**Figure 5:**
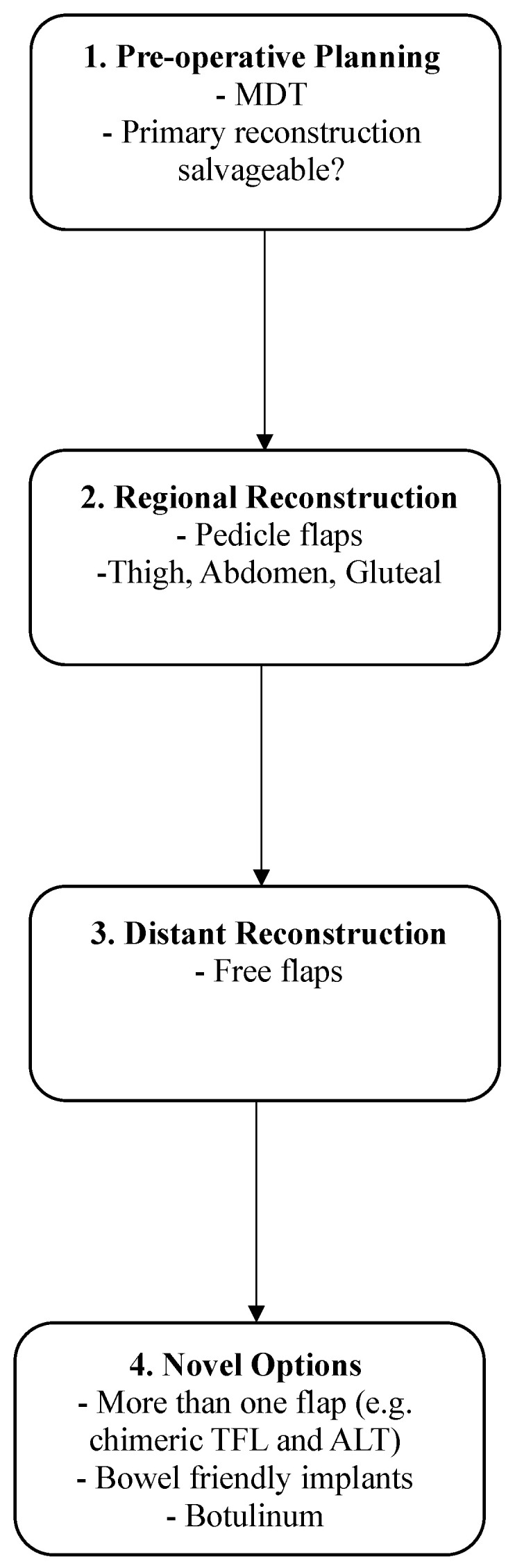
Stepwise considerations and planning for re-do reconstruction in patients undergoing second beyond TME and reconstruction.

In this paper, we described six key considerations for approaching pelvic exenteration patients. Firstly, pre-operative planning should include a dedicated MDT which includes reconstructive surgeons and a discussion on primary reconstruction and options in case a secondary reconstruction is needed. In addition, the viability and potential for re-harvesting of the primary flap needs to be explored (Figure 5). Secondly, detailed preoperative imaging, including CTA for perforator identification and DWI MRI for distinguishing between recurrent disease and post-operative anatomic distortion, may guide the reconstruction plan. Thirdly, although conventional flaps remain an option for re-do reconstruction following repeat exenteration, these options are not typically available. Fourthly, in cases where regional flaps are not available, distant free flaps such as the LD flap may provide an alternative solution. Fifthly, novel reconstructive options such as the bowel-friendly breast implant may provide viable solutions. Lastly, further development of the “Pelvic Exenteration Lexicon” to include plastic reconstructive terminology may improve communication between surgical teams and allow for more accurate data collection for reconstructive pelvic exenteration patients.

The limited number of studies reporting on outcomes following re-do reconstruction for recurrent pelvic exenterations is concerning. The large variability in the intraoperative soft tissue loss to achieve curative margin (R0) resection during pelvic exenteration, combined with the limited re-do reconstruction options, make it challenging to manage these patients. In this paper, we have proposed some key considerations that may be useful when approaching these challenging patients. However, a collaborative effort is needed to promote the development of large national databases recording outcomes and reconstructive techniques following primary and secondary pelvic exenteration with repeat reconstruction. This will allow us to improve the monitoring of short- and long-term outcomes of this cohort of patients, as well as to inform us about the reconstruction options and considerations when approaching these patients with complex wounds. This post-operative surveillance should include a combination of surgical outcomes such as the complications rate, as well as health-related quality of life outcomes, to allow for a holistic understanding of this patient cohort. Further research exploring outcomes in this cohort of patients should also aim to include detailed information on primary and re-do reconstruction. This will allow for an improved understanding of the decision-making process in deciding how to approach this complex cohort of patients. 

## 5. Conclusions

Re-do reconstruction following repeat pelvic exenteration for recurrent CRC is challenging and greatly depends on the degree of resection to achieve clear margins (R0) and a choice of primary reconstruction. Currently, there is a lack of systematic reporting of the re-do reconstruction options and outcomes, which highlights the need for future studies to examine this patient cohort in more detail. In this paper, we have provided some key considerations on re-do reconstructive options following repeat exenteration for recurrent CRC. These include the importance of a dedicated planning MDT with the input of an experienced reconstructive surgeon. The most important step in planning is to determine if the primary reconstruction is salvageable or can be re-harvested. In addition, the use of pre-operative imaging such as CT angiography and MRI may provide useful information about the regional blood supply, especially in complex cases which have required extensive resection and may therefore have distorted regional anatomy. In cases where the initial flap lacks suitability for re-harvesting, the next feasible option involves regional flaps, followed by free flaps as a last resort. In some cases, a combination of those options may be needed in order to ensure adequate coverage. Overall, this study highlights the need for a collaborative effort to promote the development of national databases to allow for improved outcome reporting for this cohort of patients.

## Data Availability

Not applicable.

## Supplementary Materials

The following supporting information can be downloaded at: https://www.mdpi.com/article/10.3390/jcm13051228/s1, PRISMA 2020 Checklist. Reference [34] is cited in Supplementary Materials.
